# Relationship between Alzheimer's disease polygenic risk score and the effects of multidomain lifestyle intervention: the SUPERBRAIN‐MEET subanalysis

**DOI:** 10.1002/alz70860_107759

**Published:** 2025-12-23

**Authors:** Seong Hye Choi, So Young Moon, Jee Hyang Jeong, Jiyun Hwang, Joohon Sung

**Affiliations:** ^1^ Inha University College of Medicine, Incheon, NA, Korea, Republic of (South); ^2^ Ajou University School of Medicine, Suwon, Gyeonggi‐do, Korea, Republic of (South); ^3^ Ewha Womans University Seoul Hospital, Ewha Womans University College of Medicine, Seoul, Korea, Republic of (South); ^4^ Graduate School of Public Health, Seoul National University, Seoul, NA, Korea, Republic of (South)

## Abstract

**Background:**

Multidomain intervention demonstrated beneficial effects on the cognition of patients with mild cognitive impairment (MCI) in the South Korean study to prevent cognitive impairment and protect brain health through multidomain interventions via face‐to‐face and video communication platforms in MCI (SUPERBRAIN‐MEET). We aimed to investigate whether the polygenic risk of Alzheimer's disease (AD) modifies the effects of a multidomain intervention in MCI patients and whether these effects differ based on APOE ε4 allele carrier status through subgroup analysis.

**Method:**

We calculated the percentile rank of polygenic risk score (PRS) of each participant (*N* = 284; intervention group: *N* = 149, control group: *N* = 135) using genome data from the Korean general population, which represents genetic predisposition to AD standardized within the Korean population. The outcome variable was defined as the percentage change from baseline in the total scale index score of the Repeatable Battery for the Assessment of Neuropsychological Status (RBANS). We assessed whether the percentile change in the RBANS total scale index score varied by PRS using linear regression analysis, adjusting for age and sex, and incorporating an interaction term between intervention status and PRS.

**Result:**

Overall, the change in the RBANS total scale index score decreased as PRS increased. When comparing the changes in the RBANS total scale index score between the intervention and control groups across different PRS levels, the intervention effect was more pronounced in the high‐PRS group (low tertile: β = 4.59, *p* = 0.069; medium tertile: β = 2.9, *p* = 0.2; high tertile: β = 5.93, *p* = 0.008). In the subgroup analysis of APOE ε4 carriers and non‐carriers, the effect of the multidomain intervention on the change in the RBANS total scale index score increased with rising PRS, particularly among APOE ε4 carriers (interaction *p* = 0.04).

**Conclusion:**

Our study demonstrates that the effectiveness of multidomain interventions may be influenced by an individual's genetic risk for AD. Specifically, the multidomain lifestyle intervention appears to be more beneficial for individuals with a higher genetic risk of AD, particularly APOE ε4 carriers with a high PRS.

